# Amiodarone induced thyroid dysfunction: A high cumulative incidence in a nationwide cohort study in Iceland

**DOI:** 10.1111/joim.20115

**Published:** 2025-07-10

**Authors:** Páll Guðjónsson, Ari J. Jóhannesson, Elías Eyþórsson, Karl Andersen

**Affiliations:** ^1^ Department of Medicine Landspitali – The National University Hospital of Iceland Reykjavík Iceland; ^2^ Faculty of Medicine, School of Health Sciences University of Iceland Reykjavík Iceland

**Keywords:** amiodarone, hypothyroidism, prognosis, thyroid diseases, thyroidectomy, thyrotoxicosis

## Abstract

**Background:**

Amiodarone induced thyroid dysfunction (AITD) is divided into amiodarone induced thyrotoxicosis (AIT) and amiodarone induced hypothyroidism (AIH). The prevalence of them varies from 1.2% to 12% for AIT and 12%–17% for AIH.

**Objectives:**

To study the incidence and complications of AITD.

**Methods:**

The cohort comprised all euthyroid patients who filled their first amiodarone prescription in Iceland in 2014, 262 persons. Data were gathered with chart review, and diagnosis confirmed with thyroid function tests. The cumulative incidence accounting for death as a competing risk was estimated for AIT, AIH, and AITD with three separate Fine‐Gray models.

**Results:**

The overall incidence of AIT, AIH, and AITD was 9.2% (95% CI: 5.6%–12.7%), 13.4% (95% CI: 9.2%–17.5%), and 22.5% (95% CI: 17.4%–27.6%), respectively, and the 5‐year cumulative incidence in the same order was 19.0% (95% CI: 11.9%–25.5%), 21.8% (95% CI: 14.7%–28.2%), and 38.5% (95% CI: 30.4%–45.7%). The highest yearly incidence rate of AIT was 9.8% during the third treatment year, and for AIH, it was 9.8% during the first year of treatment. The complications of AIT were hypothyroidism (8%), thyroidectomy (8%), hospitalizations (36%), and death (4%). Most patients (91.7%) with AIH were placed on thyroid replacement therapy.

**Discussion:**

Nearly 40% of patients taking amiodarone for 5 years acquire thyroid dysfunction, which is higher than previously described. Frequent monitoring of thyroid function should be considered during the high‐risk periods of the first and third treatment years.

## Graphical abstract

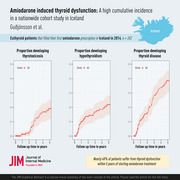

**Abbreviations**
A.fib/flutter: atrial fibrillation or flutter
AIH: amiodarone induced hypothyroidism
AITD: amiodarone induced thyroid dysfunction
AIT: amiodarone induced thyrotoxicosis
AIT1: amiodarone induced thyrotoxicosis of type 1
AIT2: amiodarone induced thyrotoxicosis of type 2
CFDS: color flow Doppler sonography
DEA: desethylamiodarone
ETA: European Thyroid Association
fT3: free triiodothyronine
fT4: free thyroxine
ScAIH: subclinical hypothyroidism
TRAb: thyroid stimulating hormone receptor antibodies
TSH: thyroid stimulating hormone

## Introduction

Amiodarone is a class III anti‐arrhythmic drug commonly used to treat cardiac arrhythmias such as atrial fibrillation, ventricular fibrillation, and ventricular tachycardia [1]. It can cause various side effects ranging from mild, such as photosensitivity of the skin and corneal microdeposits, to severe, which include peripheral neuropathy, pulmonary fibrosis, liver cirrhosis, and malignant arrhythmias [2, 3]. Thyroid‐related side effects are one of the most common adverse effects and are divided into amiodarone induced thyrotoxicosis (AIT) and amiodarone induced hypothyroidism (AIH).

AIT is divided into two subtypes and a mixed form based on the underlying pathophysiology [4]. In AIT type 1 (AIT1), there is an underlying functional autonomy, most commonly multinodular goiter or, less commonly, Graves’ disease, the former made worse and symptomatic by the high iodine load induced by amiodarone [5]. AIT type 2 (AIT2) is a destructive thyroiditis that causes the thyroid follicles to release stored thyroid hormones into the bloodstream. The time of onset differs between the two subtypes. AIT1 has a median onset time of 3.5 months, whereas AIT2 has a pattern of delayed onset with a median time of 24–32 months [5, 6]. The prevalence of AIT varies in different reports from 1.2% to 12%. It is more common in younger patients and in regions with low iodine intake, and the risk increases with increasing cumulative dosage of amiodarone [7, 8, 9, 10].

AIH can arise from the high iodine content of the drug (a 200 mg tablet contains a 50‐fold daily recommended dose of iodine) [11]. Hypothyroidism most often develops during the first treatment year [6] and is thought to arise from failure to escape from the inhibitory effects of iodine on the thyroid gland due to underlying thyroid disease, most commonly Hashimoto's disease [12]. During the first 60–90 days of amiodarone treatment, transient change of blood tests with slightly elevated thyroid stimulating hormone (TSH) with low free thyroxine (fT4) and free triiodothyronine (fT3) can be expected [11, 13]. The prevalence of AIH among amiodarone‐treated patients is reported to be 12%–17% but can be as high as 25% if cases of subclinical hypothyroidism (ScAIH) are included [14].

Iceland has a single unified electronic health record and prescription drug system, which offers a unique opportunity to undertake a population‐based study on the incidence and complications of amiodarone induced thyroid dysfunction (AITD). Therefore, we decided to undertake such a study in Iceland.

## Methods

The study's population included all euthyroid patients who filled their first amiodarone prescription in Iceland in 2014. Data on filled prescriptions were obtained from the National Prescription Medicines Register of the Directorate of Health, which contains information on all pharmaceutical prescriptions in Iceland. Individuals were followed up with a chart review from their first amiodarone prescription until death, discontinuation of amiodarone, or the end of the study period, which was defined from the 1st of January 2014 until the date of chart review (September 2019 to November 2020). Individuals who were not documented to be euthyroid before receiving amiodarone were excluded from the study.

AIT was defined as a low TSH (<0.30 mIU/L) and an elevated fT4 (>22.0 pmol/L) and/or fT3 (>6.8 pmol/L) with other causes of thyrotoxicosis being excluded. It was subclassified into AIT1, AIT2, and unknown. It was classified as type 1 if any of the following criteria were met or type 2 if none were present: (1) positive TSH receptor antibody (thyroid stimulating hormone receptor antibodies [TRAb]), (2) hypervascularity on color flow Doppler sonography (CFDS), (3) evidence of thyromegaly (moderate or greater) on CFDS, (4) one or more nodule(s) ≥10 mm on CFDS, or (5) increased scintigraphy uptake. If auto‐immune antibodies or a thyroid ultrasound were missing, the type was defined as unknown.

AIH was based on a clinical diagnosis of hypothyroidism with an elevated TSH (>4.2 mIU/L) at least 90 days after starting amiodarone. They were further classified into ScAIH if fT4 levels were normal (12.0–22.0 pmol/L) or overt AIH if fT4 levels were low (<12 pmol/L). AITD was defined as a diagnosis of either AIH or AIT. Hypothyroidism following AIT, including hypothyroidism after thyroidectomy, was classified as a complication of the thyrotoxicosis but not as AIH.

### Statistical analysis

Cumulative incidence accounting for death as a competing risk and a subdistribution hazard ratio for dose, age, and gender were estimated with three separate Fine‐Gray models for each of the disease states; AIT, AIH, and AITD, by using the *tidycmprsk, ggplot2*, *and survminer* packages in R version 4.3.2 [15, 16, 17]. Individuals were censored at diagnoses of the respective thyroid disease as well as death and discontinuation of amiodarone treatment. The number of censored cases differs among disease types, and for that reason, three distinct models were preferred over one to provide more accurate estimates of cumulative incidence for each disease.

## Results

In total, 279 individuals initiated amiodarone treatment in the calendar year 2014. Of those, 17 were not euthyroid when starting amiodarone and were excluded from the study. The remaining 262 comprised the study's population. The mean age was 72.1 years (SD, 1.3), with a mean treatment duration of 2.2 years (SD, 0.26). The mean dosage was 193 mg daily (SD, 7.2), and 71% of the population were male (Table 1).

**Table 1:** Baseline characteristics and reasons for amiodarone discontinuation.

Baseline characteristics	(*n* = 262)
Mean age—years (SD)	72.1 (1.3)
Male sex—no. (%)	186 (71.0)
Mean treatment duration—years (SD)	2.2 (0.26)
Daily dose—no. (%)	
Less than 200 mg	40 (15.3)
200 mg	209 (79.8)
More than 200 mg	13 (5.0)
Mean daily dose—mg (SD)	193 (7.2)
Still on amiodarone at the end of follow‐up—no. (%)	43 (16.4)
Stopped amiodarone and reasons for amiodarone discontinuation—no. (%)	219 (83.6)
Thyrotoxicosis	13 (5.9)
Hypothyroidism	5 (2.3)
Died during treatment	56 (25.6)
Ineffective treatment	39 (17.8)
Stable disease	23 (10.5)
Ablation	12 (5.5)
Non‐thyroid side effects	22 (10.0)
Temporary treatment after cardiac surgery	37 (16.9)
Other/unclear reason	12 (5.5)

**Fig. 1:**
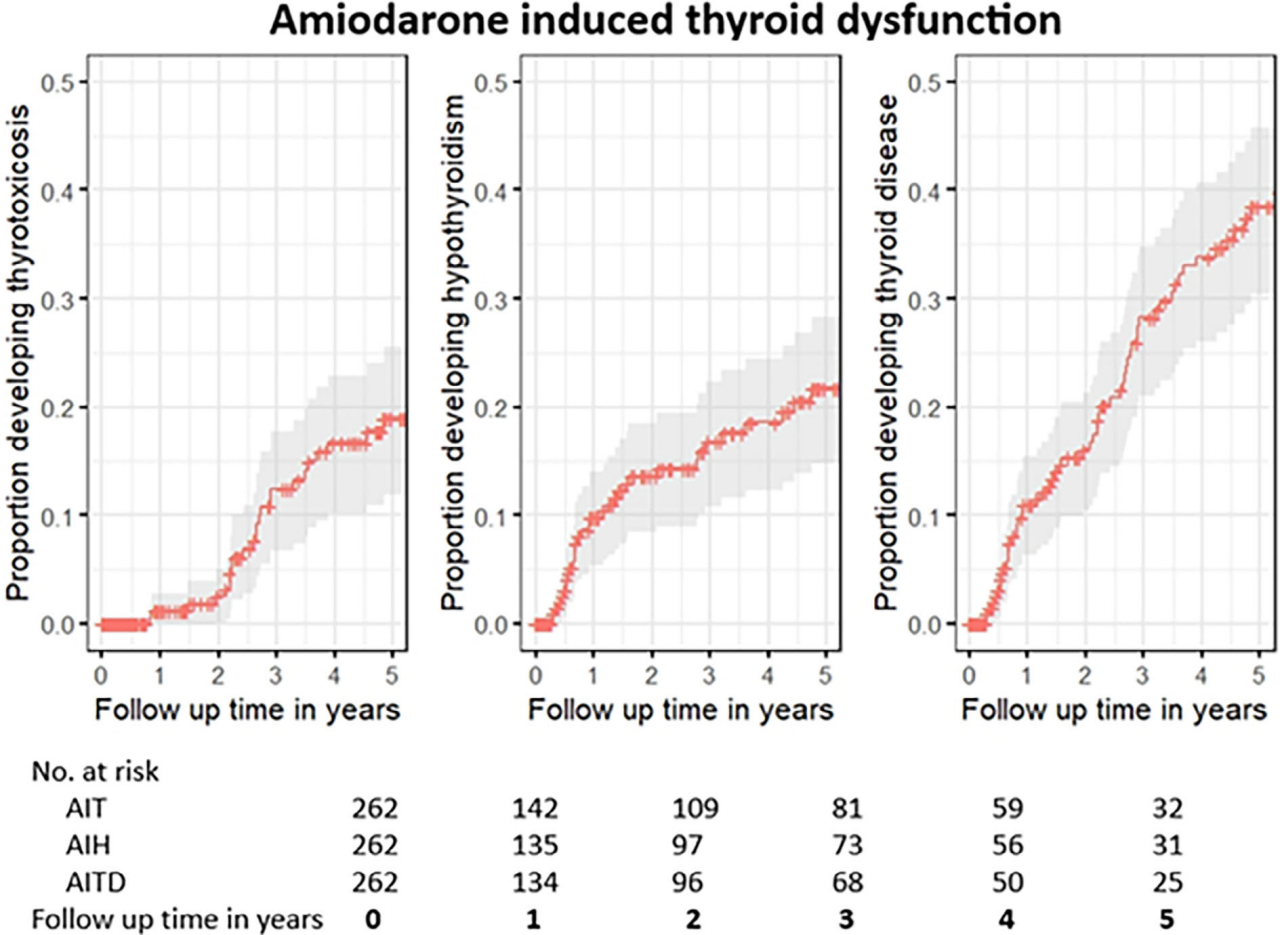
The cumulative incidence, accounting for death as a competing risk, of amiodarone induced thyrotoxicosis (AIT), amiodarone induced hypothyroidism (AIH) and amiodarone induced thyroid dysfunction (AITD) on y‐axis and time in years on x‐axis. The risk table shows the number at risk at the start of each treatment year.

AIT was diagnosed in 24 patients (9.2% (95% CI: 5.6%–12.7%)), of whom 20 were male (83.3%) and 4 (16.7%) female. It occurred after a median of 2.7 (IQR, 2.2–3.3) years, and the mean age at diagnosis was 66.0 years (SD, 4.7). The 5‐year cumulative incidence of AIT was 19.0% (95% CI: 11.9%–25.5%) (Fig. 1), and the highest yearly incidence, 9.8% (95% CI: 6.8%–12.8%), occurred during the third treatment year (Table 2). Increasing age reduced the cumulative incidence of AIT, HR 0.46 (95% CI: 0.32–0.66), whereas a higher dose of amiodarone increased it, HR 2.0 (95% CI: 1.1–3.5). Gender had a neutral effect, HR 0.7 (95% CI: 0.4–4.0).

**Table 2:** The yearly (YI) and cumulative incidence (CI) accounting for death of amiodarone induced thyroid dysfunction (AITD), amiodarone induced thyrotoxicosis (AIT), and amiodarone induced hypothyroidism (AIH) with subclassification into overt and subclinical AIH.

	Treatment year				
	1	2	3	4	5
**AIT**	
Yearly incidence, *n* (%)	2 (1.2)	2 (1.4)	13 (9.8)	5 (4.4)	2 (2.2)
Cumulative incidence, % (95% CI)	1.2 (0–2.8)	2.6 (0–5.0)	12.4 (6.8–17.8)	16.8 (10.2–22.8)	19.0 (11.9–25.5)
**AIH**					
Yearly incidence of overt AIH, *n* (%)	11 (6.3)	5 (3.3)	3 (1.6)	2.0 (2.0)	1.0 (1.0)
Yearly incidence of subclinical AIH, *n* (%)	7 (3.8)	1 (0.7)	1 (1.0)	0 (0)	2 (2.6)
Yearly incidence of AIH, *n* (%)	18 (9.8)	6 (3.8)	4 (3.2)	2 (1.8)	3 (3.2)
Cumulative incidence of AIH, % (95% CI)	9.8 (5.5–14.1)	13.6 (8.4–18.5)	16.8 (10.9–22.3)	18.6 (12.3–24.4)	21.8 (14.7–28.2)
**AITD**					
Yearly incidence, *n* (%)	20 (11.0)	8 (5.1)	17 (12.2)	7 (5.5)	5 (4.7)
Cumulative incidence, % (95% CI)	11.0 (6.5–15.5)	16.1 (10.4–21.3)	28.3 (21.0–34.8)	33.8 (26.1–41.8)	38.5 (30.4–45.7)

For subclassification of AIT, TRAb was measured in 12 patients, and none had a positive test. Ultrasound of the thyroid was performed in 11 patients, and 2 underwent technetium‐99m‐sestamibi uptake and scintigraphy. Histological diagnosis was included for the two patients who underwent thyroidectomy. Eight had sufficient data for subclassification, and all of them were classified as AIT2. They had a median time of onset after 3.1 (IQR, 2.5–3.6) years of treatment.

Treatment of AIT included amiodarone discontinuation in 12 (50%) patients. In five cases (20.8%), the treatment had been stopped before diagnosis of AIT with a median interval of 278 days (range, 51–410), whereas seven (29.2%) remained on treatment. In patients still using amiodarone at diagnosis of AIT, amiodarone withdrawal was carried out in 3 out of 9 (33.3%) with ventricular arrhythmias as the indication of treatment compared with 9 out of 10 (90%) if the indication was atrial fibrillation or flutter (A.fib/flutter). The pharmacological treatment for AIT consisted of glucocorticoid monotherapy in five patients, thionamide monotherapy in another five, and a combination therapy in eight patients, whereas six received neither treatment. Further comparison of the groups can be seen in Table 3.

**Table 3:** Comparison of complications after stopping or continuing amiodarone therapy at diagnosis of amiodarone induced thyrotoxicosis (AIT).

Comparison of stopping or continuing amiodarone in AIT				
	Amiodarone treatment			
	Stopped, *n* (%)	Stopped before AIT, *n* (%)	Continued, *n* (%)	Total, *n* (%)
**Complications**				
Hypothyroidism, *n* (%)	3* (25)	1 (20)	0	4((17)
Admission, *n* (%)	2 (17)	4 (80)	3 (43)	9 (38)
Thyroidectomy, *n* (%)	0	1 (20)	1 (14)	2 (8)
Death, *n* (%)	1 (8)	0	0	1 (4)
No complications, *n* (%)	8 (67)	0	4 (57)	12 (50)
**Age (y), mean (SD)**	64.0 (11)	63.8 (5.6)	61.7 (15)	63.3 (11.3)
**Free thyroxine, median pmol/L (range)**	43.2 (27–75)	45.4 (42–100)	35.4 (25–92)	42.9 (25–100)
**Medical treatment**				
Glucocorticoid monotherapy	5 (42)	0	0	5 (21)
Thionamide monotherapy	2 (17)	1 (20)	2 (29)	5 (21)
Both	2 (17)	4 (80)	2 (29)	8 (33)
None	3 (25)	0	3 (43)	6 (25)
**Indication**				
A.fib/flutter, *n* (%)	9 (75)	4 (80)	1 (14)	14 (58)
VT/VF, *n* (%)	3 (25)	1 (20)	6 (86)	10 (42)
**Time to normalization of TSH, median days (range)**	116 (27–325)	95 (81–173)	97 (33–179)	97 (27–325)
Total, *n*	12	5	7	24

*Two cases of hypothyroidism were transient. Abbreviations: A.fib/flutter, atrial fibrillation or flutter; VF/VT, ventricular fibrillation or ventricular tachycardia.

The complications of AIT were hypothyroidism in two patients (8%); in additional two, it was transient, and it occurred post thyroidectomy in two. Nine (36.0%) were admitted to hospital with complications related to the toxicosis, and one patient died (4.0%) from heart failure. The hospitalizations were attributable to heart failure, atrial fibrillation, weakness, and post thyroidectomy, with two cases each, and the ninth admission was because of angina.

AIH occurred in 35 patients (13.4% (95% CI: 9.2%–17.5%)), of whom 25 (71.4%) were male and 10 (28.6%) were female. Overt hypothyroidism was seen in 24 (9.2%) cases and subclinical in 11 (4.2%). The mean age at diagnosis of AIH was 73.6 years (SD, 4.8), and it occurred after a median of 0.92 years (IQR, 0.57–2.8) of treatment. The 5‐year cumulative incidence of AIH was 21.8% (95% CI: 14.7%–28.2%) (Fig. 1) and the highest yearly incidence, 9.8% (95% CI: 5.4%–14%), occurred during the first year of treatment (Table 2). Age, sex, and amiodarone dose were not significantly associated with the cumulative incidence of AIH, with the corresponding hazard ratios of 1.1 (95% CI: 0.8–1.6), 1.1 (95% CI: 0.5–2.6), and 1.3 (95% CI: 0.5–3.3), respectively. Thyroid replacement therapy was started in all patients with overt hypothyroidism, and amiodarone was stopped in three patients (8.6%). In the ScAIH group, eight (72.7%) patients received thyroxine, amiodarone was withdrawn in two (18.1%) patients, and one (9.1%) continued amiodarone without receiving treatment.

Overall, thyroid dysfunction developed in 59 individuals (22.5% (95% CI: 17.4%–27.6%)) with a 5‐year cumulative incidence of 38.5% (95% CI: 30.4%–45.7%) and the highest yearly incidence, 12.2% (95% CI: 10.6%–13.5%), during the third treatment year (Fig. 1).

## Discussion

In this nationwide chart review cohort study of all patients starting amiodarone in Iceland in 2014, we found that 38.5% of patients taking amiodarone develop thyroid dysfunction within 5 years of treatment initiation. Fig. 1 demonstrates how the rise in cumulative incidence of AITD is almost constant during the first 4 years, AIH accounting for most cases during the first 2 years and AIT for most cases in the latter two. The cumulative incidence rates are higher than previously reported. A German study reported a 10‐year cumulative incidence of 38% [8], and a Danish study reported a 5‐year cumulative incidence accounting for death as a competing risk of 24.5% in patients taking >175 mg daily [18]. Both used diagnostic codes to identify individuals with thyroid disease that could, in part, explain lower incidence rates if any cases were lost due to coding errors. A chart review study from the United States based on 169 patients estimated the 5‐year cumulative incidence of AIT to be around 20%, compared with 19% in our study, but could be slightly overestimated as it did not assess for the competing risk of death [19]. Iceland is considered an iodine‐rich area that is usually associated with a lower incidence of AIT and higher of AIH. This was not reflected in our study. Other factors that could influence the high incidence are the study being nationwide, the inclusion of cases after amiodarone withdrawal, and possibly using higher doses of amiodarone as was the case when compared to the study from Ali et al. [18].

Subtyping of AIT is difficult, and only eight had all the required investigations according to the criteria for accurate subclassification, and all were classified as type 2. The delayed onset of AIT2 is not fully understood but is thought to arise from the direct cytotoxic effects of amiodarone and its even more toxic breakdown product desethylamiodarone (DEA) [20, 21]. Both chemicals are lipophilic and accumulate in various organs, including the thyroid gland [22]. The DEA/amiodarone ratio is increased in patients with thyrotoxicosis, pointing towards DEA as the main culprit [23]. Higher adiposity body proportion would dilute the concentration of these agents in the thyroid gland, which could explain the protective effect of obesity, older age, lower dosage, and being female, as previous studies have shown [6, 7, 8, 19].

The complications of AIT in our study were serious, and similar admission rates of around 30% and a mortality of up to 20% have been described in other studies [24, 25, 26]. To avoid these complications, early diagnosis and treatment are paramount. However, previous investigations have demonstrated that surveillance of amiodarone treatment is suboptimal [27, 28, 29, 30]. Pharmacist‐led amiodarone monitoring systems can improve surveillance [31], and it can be further enhanced by applying computerized monitoring systems [32, 33]. These methods should be considered when possible, with more frequent monitoring during high‐risk periods such as the first and third treatment years for AIH and AIT, respectively.

The treatment effect of discontinuation of amiodarone or different medical therapies is difficult to interpret in retrospective studies. There is a selection bias in our groups, most clearly demonstrated by the different medical therapies that they receive and how treatment indication influences the decision to discontinue amiodarone treatment. Only 10% of patients with A.fib/flutter as the indication of treatment continued amiodarone compared with 67% if the indication was ventricular arrhythmia (Table 3). The sample size is too small to correct for these factors, making it unwise to draw any conclusions from the results.

Despite the high cumulative incidence rate, the prevalence of overt AIH in our study (9.2%) is low compared to findings in a recent meta‐analysis (14%) where the authors only considered overt hypothyroidism [14]. Low prevalence could be explained by two methodological factors. First, six cases of hypothyroidism were considered a consequence of AIT and were not included. If they were included as AIH, then four cases (1.5%) of overt hypothyroidism would have been added to the prevalence. Second, further 4.2% were clinically diagnosed with and treated for AIH despite subclinical values. This may reflect early treatment of patients that were destined to progress to overt AIH, thereby lowering its occurrence.

Cases of ScAIH were included as AIH in our study, thus adding to the high cumulative incidence of AIH and AITD. Studies that depend on diagnostic codes for finding cases of AIH, for example, the Danish and German studies above, include them, while studies relying on laboratory tests for diagnosis of AIH, for example, the previously mentioned meta‐analysis, overlook them. The latter would lead to lower incidence rates. These patients are relevant both clinically and statistically because they receive treatment either by thyroid supplementation therapy or amiodarone discontinuation, in either way rendering them immune to AIH in the remaining follow‐up period of the study. In our investigation, 72.7% of cases diagnosed with AIH despite subclinical values received thyroxine treatment, demonstrating the importance of identifying and studying this group. ScAIH is seen in up to 12%–18% of the general population, and its treatment is controversial and must be individualized [34, 35]. Unnecessary treatment can have a negative effect on survival by increasing the risk of adverse cardiac events, especially in the elderly [35, 36, 37, 38, 39].

The early onset of AIH has been demonstrated in other studies [6, 40, 41]. The high incidence in the first treatment year raises the question of whether the transient hypothyroid period at the start of treatment could be prolonged beyond 90 days in some instances. Two placebo‐controlled randomized trials showed that more than 10% of patients have elevated TSH after 3 months of treatment [41, 42], but not all required treatment. The 2018 European Thyroid Association guidelines recommend treating patients with overt AIH but evaluating ScAIH on an individual basis [43]. Subclinical elevations are very common in amiodarone patients, and this field requires more investigations to aid in clinical decision‐making.

The main strength of this study is its completeness, including all individuals diagnosed with thyroid disease during the study period having detailed information on the subjects with clinical endpoints adjudicated with chart review that should give a fair estimate of true values. By gaining information through medical records, we were able to exclude coding errors influencing the results and made it possible to exclude abnormal thyroid function tests that were not related to amiodarone treatment. It made data about complications achievable and collected the actual dates and reasons for amiodarone discontinuation. The main limitation is the small sample size with relatively few cases, which limits statistical power, making statistical comparison difficult.

We conclude that 38.5% of patients taking amiodarone for 5 years or longer acquire thyroid dysfunction. The risk is continuous during this period, with the highest risk of AIH in the first treatment year and AIT in the third treatment year. Frequent monitoring with thyroid function tests should be considered during these high‐risk periods. The high cumulative incidence and the complications of AIT are reminders to use amiodarone with care and only when other treatment options are clearly inferior.

## Ethics statement

The research was approved by the national bioethics committee of Iceland (approval no. VSN 16–024) and was conducted according to the Helsinki Declaration.

## Conflict of interest statement

The authors declare no conflicts of interest.
